# Linkage analysis of cross-sectional and longitudinally derived phenotypic measures to identify loci influencing blood pressure

**DOI:** 10.1186/1471-2156-4-S1-S26

**Published:** 2003-12-31

**Authors:** Neil Shephard, Milena Falcaro, Eleftheria Zeggini, Philip Chapman, Anne Hinks, Anne Barton, Jane Worthington, Andrew Pickles, Sally John

**Affiliations:** 1 ARC Epidemiology Unit, University of Manchester, Manchester, United Kingdom; 2 Biostatistics Group, University of Manchester, Manchester, United Kingdom; 3 Research and Development Genetics, AstraZeneca, Alderley Park, Macclesfield, United Kingdom; 4 Centre for Integrated Genomic Medical Research, University of Manchester, Manchester, United Kingdom

## Abstract

**Background:**

The design of appropriate strategies to analyze and interpret linkage results for complex human diseases constitutes a challenge. Parameters such as power, definition of phenotype, and replicability have to be taken into account in order to reach meaningful conclusions. Incorporating data on repeated phenotypic measures may increase the power to detect linkage but requires sophisticated analysis methods. Using the simulated Genetic Analysis Workshop 13 data set, we have estimated a variety of systolic blood pressure (SBP) phenotypic measures and examined their performance with respect to consistency among replicates and to true and false positive linkage signals.

**Results:**

The whole-genome scan conducted on a dichotomous hypertension phenotype indicated the involvement of few true loci with nominal significance and gave rise to a high rate of false positives. Analysis of a cross-sectional quantitative SBP measure performed better, although genome-wide significance was again not reached. Additional phenotypic measures were derived from the longitudinal data using random effects modelling for censored data with varying levels of covariate adjustment. These models provided evidence for significant linkage to most genes influencing SBP and produced few false positive results. Overall, replicability of results was poor for loci, representing weak effects.

**Conclusion:**

Longitudinally derived phenotypes performed better than cross-sectional measures in linkage analyses. Bearing in mind the sample design and size of these data, linkage results that fail to replicate should not be dismissed; instead, different lines of evidence derived from complementary analysis methods should be combined to prioritize follow up.

## Background

Lander and Kruglyak [1] proposed that very stringent significance thresholds should be achieved before declaring linkage. However, LOD scores in published whole-genome scans of complex traits have rarely fulfilled these criteria. Since the effect size of most loci in complex diseases is expected to be small, it is anticipated that true susceptibility loci will achieve only modest levels of significance in the sample sizes of many current studies. As there are usually insufficient resources to follow up all loci with modest LOD scores, it is crucial to select those most likely to contain true susceptibility loci. Replication of linkage peaks across independent data sets is a commonly used strategy to confirm true loci. However, there are still very few examples of linkage peaks that are replicated uniformly. Phenotype definition, low power to detect small genetic effects, type I error, and genetic heterogeneity all contribute to inconsistent results.

Phenotype definition is a critical factor that affects the ability to detect trait loci. The simulated data from Genetic Analysis Workshop 13 (GAW13) provide enough clinical and environmental information collected over time to generate several potential phenotypes aimed at detecting loci influencing systolic blood pressure (SBP). Incorporating data on repeated phenotypic measures may increase the power to detect linkage and will be the only way to detect genes that influence variation in traits over time. Obtaining longitudinal data is far more resource-intensive than collecting cross-sectional data. It is, therefore, important to know whether using longitudinal phenotypes does have more power than cross-sectional phenotypes. The GAW13 simulated data therefore provide an excellent opportunity to compare the ability of cross-sectional with longitudinal phenotypic measures to detect linkage to known loci.

Our aim was to explore the benefits in terms of power, scientific insight, and replicability of linkage analysis findings of a model-based approach to phenotype characterization. Several different phenotypic models of SBP from the GAW13 simulated data were considered, including a dichotomous hypertension phenotype, a cross-sectional measure of SBP and longitudinal measures of 'trait' SBP, adjusted for various sets of covariates. Evidence for linkage for each phenotypic measure was examined. Irrespective of the phenotype studied, it is still considered important to replicate evidence of linkage in an independent data set. Therefore, we analyzed three replicates to examine the consistency of results. All analyses were done blind to the simulation conditions, which were provided at the workshop.

## Methods

### Pedigrees

Replicates 4, 10, and 21 were used in all analysis. Families with more than 20 individuals were excluded from the data set to reduce computational time. Two hundred and seventy seven pedigrees were analyzed with a mean of 11 members (minimum 7 and maximum 20 members). Of the 3155 subjects in the pedigrees, 33% had genotype data available.

### Systolic blood pressure phenotypes

Six phenotype models describing SBP were defined; hypertension, a cross-sectional measure of SBP, and four models derived from longitudinal data, Models 1–4. Absence or presence of hypertension was used as a qualitative phenotype; individuals were defined as affected if they had a diagnosis of hypertension at any examination. Eighty-five families, containing enough affected members with genotype data, contributed to linkage analysis in each of the three replicates.

Taking the SBP value at the first examination for each subject generated a cross-sectional measure of systolic SBP. No one in replicates 4, 10, or 21 was recorded as receiving antihypertensive treatment at the first time interval; therefore, no adjustment for treatment was necessary.

Longitudinal SBP data were analyzed with a subject-specific approach (e.g., ref [2]). All systolic BP measures taken over time were included in the analysis. However, some subjects on some occasions were receiving antihypertensive medications. Since their recorded systolic BP was lower than it would have been if they had not been on treatment, these observations were treated as censored. Therefore, to account for the presence of both repeated measurements and right censoring, a mixed probit-normal or Tobit model [3] with a subject-specific random intercept was estimated using the program GLLAMM (generalized linear latent and mixed models) in STATA 7 [4].

For an untreated SBP measurement *y*_*it *_made on individual *i *at time *t *the model was specified as *y*_*it *_= β*X*_*it *_+ *u*_*i *_+ *e*_*it*_, where *X*_*it *_is a vector of covariates (including a constant) that may vary over time, β is a vector of regression coefficients, *u*_*i *_is a N(0, σ_u_^2^) subject-specific random effect, *e*_it _are N(0, σ_e_^2^) disturbance terms with corr(u,e_._) = 0 for all *t *and corr(e_.s_,e_.t_) = 0 for *s *≠ *t*. For censored observations Pr[Y >*y*_*it*_] = Φ ((β*X*_*it *_+ *u*_*i *_- *y*_*ij*_) / σ_*e*_,1), where Φ(.,1) is the standard normal cumulative density function.

The empirical Bayes' estimates of the individual random effects (the subject level residuals {}) were extracted for use as adjusted longterm SBP phenotypes for input to SOLAR [5]. Fitted to males and females separately, four different adjustments for covariates were considered: Model 1: age, age squared and body mass index; Model 2: covariates in Model 1 plus cohort; Model 3: covariates in Model 2 plus smoking and alcohol consumption; Model 4: covariates in Model 3 plus cholesterol level and fasting glucose. Covariates were selected for inclusion in a step-wise manner, starting with subject-specific factors known to strongly affect SBP, then an allowance for any possible cohort effect, followed by further environmental covariates. Finally, we included possible intermediate phenotypes that contribute to the variation in SBP, including such covariates may reduce the power to detect linkage for genes contributing to these intermediate phenotypes.

The models for repeated censored data yielded consistent covariate effects across replicates for cigarettes per day and the absence of effect for alcohol, moderate consistency for cholesterol, and weak consistency for glucose.

### Heritability and linkage analysis

Heritability estimates were obtained using variance components analysis as implemented in the SOLAR package [5]. Multipoint quantitative linkage analyses were conducted on the cross-sectional SBP phenotype and the standardized residuals for the longitudinal systolic SBP values.

GENEHUNTER version 2 [6] was used to carry out a nonparametric linkage (NPL) analysis of the whole genome, treating hypertension as a qualitative trait, as described above.

LOD scores > 1 were considered as nominal evidence of linkage, LOD scores > 2.2 as suggestive evidence, LOD scores > 3.6 as genome-wide evidence and LOD scores > 5.4 as confirmed linkage.

### Pedigreed disequilibrium test (PDT) analysis

Microsatellite markers under areas of increased allele sharing (*p *< 0.1) were tested for evidence of association with the hypertension phenotype. The PDT method [7] was used, as this is a test for linkage and association in general pedigrees and can currently only be applied to qualitative data. In total 99 markers were analyzed.

## Results

### Hypertension phenotype

No NPL scores achieving genome-wide significance were detected using hypertension as a trait. All replicates produced linkage peaks with a NPL score > 1. The most significant results were on chromosomes 15 (109 cM), 18 (28–70 cM) and 21 (23–50 cM) for replicates 4, 10, and 21, respectively. The former two loci did not contain true trait loci. There was very little consistency between replicates, with only chromosome 21 showing some evidence of linkage in more than one replicate. Table 2 shows maximum LOD scores achieved for genes affecting SBP.

### SBP phenotypes models

All of the phenotypic measures of SBP were highly heritable (>70%) and are listed in Table 1. All phenotypic measures of SBP produced LOD scores > 1 for all replicates. The maximum LOD scores achieved for each trait at each gene locus are shown in Table 2. All but one of the loci influencing SBP was detected in at least one replicate and in general evidence for linkage was greater using the longitudinally derived phenotypes. The genes Gb35 on chromosome 13 and Gs11 on chromosome 15 were only detected by the longitudinal phenotypes.

There was little overall consistency in loci detected between replicates as shown in Table 3. The linkage region on chromosome 21 (20–50 cM) was the only region that showed evidence of linkage in all three replicates. Genes Gb35 on chromosome 13 and Gs11 on chromosome 15 were only detected in a single replicate (4 and 21 respectively).

### PDT analysis

To provide further evidence of linkage for potential linkage regions, microsatellite markers mapping to weak regions of linkage on chromosomes 2, 5, 10, 11, 13, 15, 16, 20, and 21 were tested for evidence of association to hypertension using the PDT method. Seven markers showed evidence of preferential transmission (*p *< 0.05) using the sum and/or average PDT result. Of these, two mapped to true disease loci near Gb35 and Gs12.

## Discussion

Six phenotypic measures of SBP were generated for the purposes of this analysis. The two cross-sectional measures required only one data point per subject. Incorporating all longitudinal data is likely to increase the power to detect linkage, but there are several possible methods that could be applied to derive longitudinal phenotypes. The four adjusted 'trait' SBP measures derived in this analysis were based on all the available SBP measures for each individual and were estimated as subject-specific random intercepts using the program GLLAMM. This approach is appealing because it allows adjustment for a treatment effect to be included in the model and, by sequentially including covariates, the effect of different covariate adjustments on linkage results can be observed. Estimation is by maximum likelihood using adaptive quadrature, an approach that has good properties for a wide range of measures and sampling schemes, is very flexible, and not especially computationally demanding (a few minutes on a personal computer).

The categorical hypertension phenotype showed the least evidence of linkage to any of the true loci. Two of the most significant regions of linkage identified using hypertension as a qualitative trait (chromosomes 15 and 18 for replicates 4 and 10, respectively) only showed any evidence for linkage in a single replicate and were not consistent with any other measure of SBP. These loci are in fact false positive results. The relatively poor performance of the hypertension phenotype may, however, be related to the general population sample design that yielded a rather small effective sample size (85 families per replicate). To overcome the loss of power inherent to such a study design, the PDT method was employed to provide complementary evidence toward the involvement of the implicated chromosomal regions. The quantitative cross-sectional measure derived from SBP at first exam detected three genes at suggestive evidence of linkage, one with a LOD score 1.8, and produced three false positive results on chromosomes 5, 8, and 12. None of the LOD scores reached genome-wide significance and there was little consistency between replicates. The longitudinally derived phenotypes were superior to the cross-sectional measure both in terms of the number of loci detected and the magnitude of the evidence for linkage. These phenotypes were able to detect five loci influencing SBP at suggestive evidence of linkage, four of these at a genome-wide significance level. In general, LOD scores also improved with adjustment for covariates, although this was not always the case, suggesting that using all the models provided the most information about linkage at any given locus.

Because this analysis was performed blind to the answers, it was not possible at the time to determine which linkage peaks corresponded to true trait loci. This made the initial interpretation of the results difficult because many of the LOD scores were modest and there were inconsistencies in results between replicates. Deciding which loci are true or false is a difficult problem for researchers when faced with real whole-genome scan results. We therefore prioritized loci more likely to be true using the following criteria: evidence of linkage in more than one replicate, evidence of linkage with more than one phenotype, and evidence of linkage and association with a nearby microsatellite marker. Having followed this process, we concluded that we would suggest that chromosome 21 harbors at least one locus influencing BP and we would predict that the regions on chromosomes 5, 11, and 13 would also be worthy of further investigation. Interestingly, the chromosome 21 loci, detected through the use of each of the phenotypic measures, cumulatively accounted for 54% of the variance. We would therefore have followed up all the true loci except Gb7, which we did not detect, and Gs11, because it produced evidence for linkage in just a single replicate. We would also have followed up one false positive result (chromosome 11) and not followed up the one on chromosome 12.

The aim of using the standardized residuals from the longitudinal BP models was to increase power to detect linkage to SBP. We used a random intercept residual and did not explore using random slope residuals that might have detected genes for late onset high SBP. While such residuals are easily estimable within our approach, we considered the *a priori *scientific case for them to be less convincing. It is, therefore, perhaps surprising that we were able to detect the three slope genes influencing SBP with such high LOD scores. Two of these slope genes mapped to chromosome 21; Gb37 also mapped to the same locus as Gs12 and explained 40% of the variance of diastolic blood pressure. Because diastolic blood pressure directly affects SBP, it is not clear exactly what the linkage is picking up on chromosome 21.

## Conclusions

This analysis of the GAW13 simulated data has demonstrated that longitudinally derived phenotypes have more power to detect baseline and slope genes than cross-sectional measures of the same trait, thus suggesting that they are likely to be essential for many genetic studies. The successful identification of all but one true trait loci using GLLAMM indicates that it is an effective method for deriving powerful longitudinal phenotypes. Furthermore, the limited overlap observed among replicates suggests that unreplicated linkage results should not be lightheartedly dismissed, especially when they may represent weak effects, as is the case in complex diseases. Complementary evidence derived from diverse data analyses should, therefore, drive the prioritization of linkage peaks for follow-up, in order to maximize use of the available resources to detect real disease genes.

## Figures and Tables

**Table 1:** Heritability estimates obtained from SOLAR

	**Replicate**		
	
**Model**	**4**	**10**	**21**
SBP + covariates	87.9%	70.7%	74.6%
Model 1	80.4%	76.5%	78.3%
Model 2	79.9%	76.6%	78.2%
Model 3	75.4%	73.8%	73.0%
Model 4	76.5%	76.0%	74.9%

**Table 2:** Max LOD scores reached in any replicate for genes affecting blood pressure for each phenotypic measure of SBP. Peak LOD within 20 cM of true location, using the sex averaged map.

		**Phenotype**					
		
**Gene**	**Location**	**Hypertension**	**SBP 1^st ^exam**	**Model 1**	**Model 2**	**Model 3**	**Model 4**
Gb34	Chr 5: 176.1 cM	ND^A^	1.8	ND	ND	ND	ND
Gb35	Chr 13: 85.2 cM	ND	ND	2.8	5.2	ND	ND
Gb36	Chr 7: 47.5 cM	ND	ND	ND	ND	ND	ND
Gb37	Chr 21: 29.1 cM	1.4	2.4	9.7	9.7	11	11.1
Gs10	Chr 21: 53.6 cM	1.4	2.4	10.4	10.3	11	11.1
Gs11	Chr 15: 4.5 cM	ND	ND	2.2	2.2	1.5	2.1
Gs12	Chr 21: 29.5 cM	1.4	2.4	9.7	9.7	11	11.1
False positives		1.5 (Chr 3)	2.7 (Chr 5)	1.7 (Chr 9)	1.7 (Chr 9)		1.4 (Chr 11)
		1.6 (Chr 5)	1.5 (Chr 8)	2.3 (Chr 11)	2.3 (Chr 11)		
		1.4 (Chr 9)	3.4 (Chr 12)				
		1.6 (Chr 18)					

^A^ND: no evidence of linkage detected at LOD > 1.3.

**Table 3:** Evidence for linkage in more than one replicate for each gene affecting SBP

			Number of replicates with evidence of linkage		
			
Gene	Variance	Mean	Same phenotypic measure	Any phenotypic measure	PDT analysis
Gb34	0.25	0	1	1	0
Gb35	0.15	0	1	1	1
Gb36	0.1	0	0	0	0
Gb37^A^	0.4	0	2	3	0
Gs10	0.124	0.342	3	3	1
Gs11	0.058	0.137	1	1	0
Gs12	0.015	0.084	2	3	0

^A^The variance and mean of Gb37 is the locus effect on diastolic blood pressure.
